# Case Report of a Rare Cystic Mediastinal Lymphangioma Mimicking Recurrent Pleural Effusion

**DOI:** 10.1155/2019/1301845

**Published:** 2019-05-26

**Authors:** Fateme Salehi, Mark Landis, Richard Inculet, Daniele Wiseman

**Affiliations:** ^1^Department of Radiology, Victoria Hospital, London Health Science Centre, Western University, London, ON, Canada N6A 5W9; ^2^Division of Thoracic Surgery, Victoria Hospital, London Health Science Centre, Western University, London, ON, Canada N6A 5W9

## Abstract

Mediastinal lymphangiomas are rare benign congenital malformations, but complications can occur, including infection, cystic hemorrhage, superior vena cava syndrome, airway compromise, and chylothorax. Radiologically, lymphangiomas are well-defined masses, with low attenuation ranging from simple to complex fluid and fat. They often encase adjacent mediastinal structures. We present a case of mediastinal lymphangioma in a young female, who presented with recurrent complex pleural effusions, initially thought to represent an empyema and/or necrotic mass. Despite surgical chest tube and interventional radiology drainage, fluid reaccumulated. Upon further review, the interventional and thoracic radiologist concurred that the complex collection was in fact predominantly extra pleural in location. The patient underwent partial resection after it was discovered intraoperatively that the extra pleural cystic mass was contiguous with and extended deeply into the mediastinum. Histopathology confirmed the diagnosis of lymphangioma.

## 1. Introduction

Mediastinal lymphangiomas are rare, benign congenital malformations that arise from focal proliferation of lymphatic vessels. They comprise up to 4.5% all mediastinal tumors [1]. The most common location for lymphangiomas is neck and axilla, with only 1% being confined to the mediastinum [1]. The former group tends to present in infants and the latter more commonly occurs later in life [2]. Lymphangiomas are benign, but complications can occur including infection, cystic hemorrhage, superior vena cava syndrome, airway compromise, chylothorax, and chylopericardium [3]. Once completely resected, recurrence is reportedly rare. We present a rare case of a large cystic mediastinal lymphangioma that was resected through open thoracotomy.

## 2. Case Presentation

**Figure 1:**
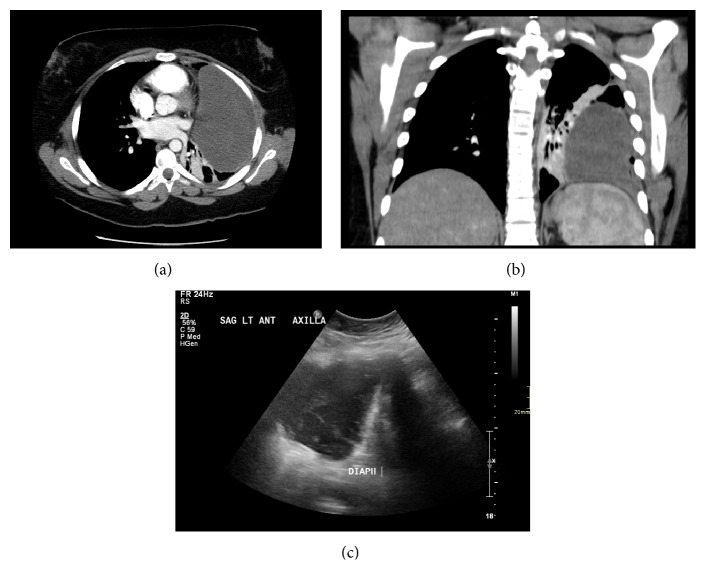
(a) Contrast enhanced axial image through upper thorax demonstrates a complex left pleural fluid collection with heterogeneous attenuation and internal septations. No vascularity was demonstrated to suggest and underlying mass. (b) Coronal image demonstrate partial collapse of the lower and upper lobes due to mass effect from the pleural collection. (c) Sonographic image through the left hemidiaphragm shows a mildly complex pleural fluid collection.

We present the case of an 18-year-old female with cystic mediastinal lymphangioma. She initially presented to a peripheral hospital with left-sided chest pain and worsening shortness of breath, and no constitutional symptoms. She was found to have a large left-sided pleural effusion on CT thorax, presumed to be parapneumonic. She was started on antibiotic therapy including cephalexin and azithromycin. Initial thoracentesis was performed and 700ml of murky blood-tinged fluid drained. A 10.2 French Wayne pigtail catheter was inserted and approximately 2 liters of similar fluid was drained. Despite the chest tube, she had evidence of a persistent large, loculated left-sided pleural effusion and was transferred to our Tertiary Care Centre for further management. At presentation to our centre, she was found to have elevated white blood cell count of 15.4 x 10^9^/L and platelet count of 634 x 10^9^/L. A bedside chest tube insertion was undertaken, with subsequent CT thorax demonstrating no change in the size of the large left complex collection (Figure 1). Therefore, the presence of an underlying abscess with a possible underlying necrotic mass was considered, and interventional radiology was consulted with the view to biopsy or drain depending on context characteristics. The fluid collection appeared extra pleural to the IR upon review of the CT. The thoracic radiologist agreed. Ultrasound revealed a multiseptated rounded extra parenchymal thoracic collection. A chest tube was inserted under ultrasound and fluoroscopic guidance, with 500 ml of serosanguineous fluid drained. Cytology did not reveal malignancy. The patient was discharged on Levofloxacin. Follow-up CT in one month demonstrated accumulation of the fluid, not expected in the case of a presumed empyema after adequate drainage. The patient underwent video-assisted thoracic surgery (VATS), which was converted to open thoracotomy upon discovery of a cystic structure extending into the mediastinum. The mass was partially resected and talc pleurodesis was performed. Histopathology revealed the diagnosis of mediastinal lymphangioma, demonstrating an irregular multiloculated cyst wall composed of channels and papillae and lined by bland flat cells. Cells were positive for D2-40, CD31, and CD34. The wall was composed of mature fibrovascular and adipose tissue. The outer aspects of the wall were lined by mesothelial cells.

## 3. Surgical Findings

VATS demonstrated abundance of loose adhesion to the chest wall, which were cauterized. A large cystic structure was encountered extending anteriorly and superiorly, and the procedure was converted to posterolateral thoracotomy for better visualization and resection. The cyst was dissected superiorly to the level of the hilum and inferiorly to the diaphragm. Posteriorly, it enveloped the aorta and subclavian vessels. The phrenic nerve appeared to run through the middle of the cyst and was dissected off the cystic wall, with residual wall left along the nerve. The medial wall was unresectable due to the involvement of mediastinal structures. Talc was instilled into the chest to prevent postoperative chylothorax. Postoperatively, the patient was started on total parenteral nutrition to prevent chylothorax formation. At six-month follow-up, the patient was well, and imaging showed no recurrence.

## 4. Discussion

Our case illustrates the clinical and imaging features of mediastinal cystic lymphangioma and its management. Mediastinal cystic lymphangioma is a rare congenital malformation that often presents in the second decade [2]. Cystic lymphangiomas presumably result from sequestration of lymphatic tissue that is isolated from the lymphatic system. Lymphangiomas are classified into three histological types as simple (capillary), cystic (hygroma), and cavernous lymphangiomas. Clinically they are encountered in infants as the cavernous or cystic type that extend into the neck or occur later in life as confined mediastinal cysts [4]. Cystic lymphangiomas are characterized by the presence of large cystic spaces that are lined by endothelial cells and filled with clear proteinaceous or chylous fluid [5].

On imaging, cystic lymphangiomas appear as well-defined, lobular masses that can be associated with pleural effusions. On Computed Tomography (CT), lymphangiomas appear as cysts with a smooth contour and encase adjacent mediastinal structures. Lymphangiomas are homogenously low attenuating, with attenuation ranging from simple to complex fluid and fat [1, 6].

On MR imaging, findings include signal isointensity to muscle on T1 weighted images and hyperintensity on T2 weighted images, with variability depending on the extent of cystic components and proteinaceous contents [7].

Top differential considerations include necrotic neoplasm, thymic cyst, hematoma, seroma, or abscess, as well as mature teratoma. The diagnosis of lymphangioma in patients with typical findings of a uniform cystic mass at the anterior mediastinum is relatively evident. However, in atypical cases that exhibit no convincing evidence of cystic components or present in unusual locations, preoperative diagnosis may not be possible solely based on imaging characteristics.

In our case, imaging features were nonspecific, and the clinical finding of recurrent thoracic fluid accumulation raised suspicion for an underlying abscess initially, which did not respond to antibiotic therapy and drainage. Given its failure to result with conservative management, thoracic surgery was performed. Given its intimate association with mediastinal structures, VATS resection may not be optimal, and the recommended treatment is open thoracotomy for surgical resection of cases that extend to encase mediastinal structures [3]. Reported recurrence rate after complete surgical resection is low (6%), whereas recurrence rates of up to 35% are found in cases where resection is incomplete due to proximity to vital structures [3]. Pleurodesis with use of talc or other agents including tetracycline, minocycline, bleomycin, OK-432, and povidone iodine has demonstrated efficacy with success rates ranging from 80 to 100% [3]. In cases of refractory chylothorax, insertion of a pleuroperitoneal or pleurovenous shunt is an alternate solution [3].

We highlight that, in cases of recurrent thoracic fluid accumulation, lymphangioma should remain an unusual consideration. Gross total resection is often curative, with very low recurrence rates.
